# Anévrysme géant de l'artère pulmonaire révélant un syndrome de Behçet

**DOI:** 10.11604/pamj.2015.20.411.5941

**Published:** 2015-04-27

**Authors:** Madiha Mahfoudhi, Sami Turki

**Affiliations:** 1 Service de Médecine Interne A, Hôpital Charles Nicolle, Tunis, Tunisie

**Keywords:** Syndrome de Behçet, anévrysme, artère pulmonaire, hémoptysie, Behcet syndrome, aneurysm, pulmonary artery, hemoptysis

## Image en medicine

Des atteintes vasculaires graves notamment les anévrysmes des artères pulmonaires peuvent compliquer le syndrome de Behçet. Ces atteintes sont rarement révélatrices entrainant un retard diagnostique. Patient âgé de 32 ans, hospitalisé pour des épisodes récidivants d'hémoptysie de faible abondance. L'interrogatoire a trouvé la notion d'aphtose bipolaire récurrente évoluant depuis deux ans. L'examen physique a objectivé une apyrexie, un aphte buccal, une cicatrice d'aphte génital, des lésions de pseudofolliculite au niveau du dos et un test pathergique positif. L'examen ophtalmologique, neurologique et abdominal était sans anomalies. Sur le plan biologique, il n'avait pas de syndrome inflammatoire. Il avait une anémie normochrome normocytaire avec une hémoglobine à 10,5 g/dl. La radiographie de thorax a révélé une opacité homogène de contours réguliers para-hilaire gauche. L'angio-scanner thoracique a éliminé une origine parenchymateuse qui peut être en rapport avec une néoplasie, une vascularite ou une infection pulmonaire. Il avait plutôt un anévrysme géant au niveau de l'artère pulmonaire gauche de 10 cm de grand axe. Le diagnostic de syndrome de Behçet a été retenu devant l'association d'une aphtose bipolaire récurrente, de pseudofolliculites et un test pathergique positif. L'anévrysme de l'artère pulmonaire rentre dans le cadre d'un angio-Behçet. Il a reçu un traitement médical qui s'est basé une corticothérapie à forte dose avec diminution progressive des doses, des boli mensuels de cyclophosphamide et colchicine. Il n'a pas présenté de récidive des hémoptysies. L'anévrysme a discrètement diminué de taille. Un traitement chirurgical lui a été prévu.

**Figure 1:**
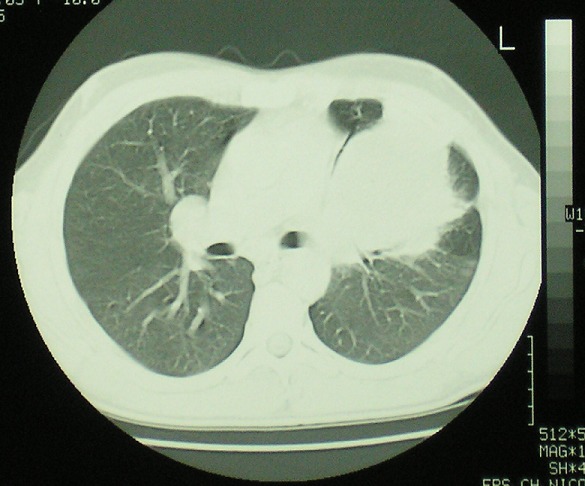
Angio-scanner thoracique: anévrysme géant de l'artère pulmonaire gauche de 10 cm de grand axe

